# Predictors of reflux persistence after endoscopic dextranomer/hyaluronic Acid copolymer injection in pediatric patients with Vesicoureteral reflux: short-term results

**DOI:** 10.1038/s41598-024-62449-6

**Published:** 2024-07-02

**Authors:** Ismail Onder Yilmaz, Nebil Akdogan, Mutlu Deger, Ibrahim Atilla Aridogan, Volkan Izol, Nihat Satar

**Affiliations:** 1 Department of Urology, Ceyhan State Hospital, Ceyhan, 01940 Adana, Turkey; 2 Department of Urology, Faculty of Medicine, Cukurova University, Adana, Turkey; 3 Private Clinic, Cukurova Urology Center, Adana, Turkey

**Keywords:** Urology, Paediatric urology

## Abstract

This study aims to investigate the factors effective in predicting the persistence of reflux after the first subureteric transurethral injection (STING) of dextranomer/hyaluronic acid copolymer in pediatric patients with vesicoureteral reflux. The data of patients without a previous history of surgery to treat vesicoureteral reflux and who underwent STING for the first time between September 2011 and November 2020 were investigated retrospectively. After considering exclusion criteria, of 199 patients, 127 patients and 180 renal units were suitable for inclusion. A renal unit-based evaluation was made. Age < 61 months (univariate: p = 0.001, multivariate: p = 0.015, HR: 2.352 (1.181–4.686), OR (95% CI)), moderate reflux level (grade 3) (univariate: p < 0.001, multivariate: p = 0.019, HR: 2.703 (1.177–6.209), OR (95% CI)), DRF (differential renal function) < 45 (univariate: p = 0.020, multivariate: p = 0.047, HR: 1.992 (1.009–3.935), OR (95% CI)), and UDR (ureteral diameter ratio) > 0.15 (univariate: p < 0.001, multivariate: p = 0.005, HR: 2.786 (1.368–5.672), OR (95% CI)) were found predictors of reflux persistence after STING surgery both univariate and multivariate analysis. High reflux level (grade 4–5) was statistically significant in univariate analysis (p < 0.001) but not statistically significant in multivariate analysis (p = 0.215). In our study, UDR and DRF were found to be factors affecting reflux persistence. UDR and DRF should be considered in order to predict reflux resolution in patients who will undergo STING.

## Introduction

Although vesicoureteral reflux (VUR) disease is one of the most common urological anomalies seen in children, its incidence is approximately 1%^1^. If untreated, recurrent urinary tract infections can lead to renal scarring^2^, hypertension^3^, and end-stage renal failure^4^. VUR is one of the most common causes of pediatric nephropathy^5^.

There are various methods in the surgical treatment of VUR, including endoscopic injection and ureteroneocystostomy (open and minimally invasive). Endoscopic treatment was first reported by Matouschek^6^ in 1981. In 1984, Puri and O’Donnell^4^ described an endoscopic subureteric transuretheral injection (STING) procedure using polytetrafluoroethylene material to treat VUR^7^. Different injection materials and endoscopic methods were represented in the following years. Many studies in the literature compare the effects of different methods and different materials on reflux resolution rates after endoscopic injections^8–11^. In a meta-analysis, renal unit-based evaluation showed that the reflux resolution rate after STING was 75.7%^8^. In other words, patients who have persistent reflux after STING will either have repeated STING requirements or require ureteroneocystostomy despite recurrent STING.

Various studies have investigated the effect of the ureteral diameter ratio (UDR) in predicting the probability of reflux resolution after STING^12–14^. However, only a limited number of studies have examined the effect of differential renal function (DRF) reduction on resolution after STING^15,16^. We aimed to investigate the effect of some parameters in predicting the resolution of reflux after a first dextranomer/hyaluronic acid copolymer injection.

## Methods

Between September 2011 and November 2020, patients younger than 18 years with vesicoureteral reflux, without a previous history of surgery for vesicoureteral reflux, and who underwent dextranomer/hyaluronic acid copolymer injection for the first time were analyzed retrospectively. Ethics committee approval of the study was obtained from the ‘Non-invasive Clinical Research Ethics Committee’ of the University of Çukurova (approval number; February 4, 2023, 130/62). The informed consent form was not obtained from the patients because the ‘Non-invasive Clinical Research Ethics Committee’ of the University of Çukurova exempts this study from the informed consent form due to its retrospective nature. Also no contact was made with patients face to face or via communication tools. The hospital automation system was used to collect the pediatric urology data of the patients. Patients with missing data and patients with accompanying pathologies, such as a ureteropelvic–ureterovesical junction obstruction, an ectopic ureter, a posterior urethral valve, a double collecting system, a spinal deformity, or a neurogenic bladder, were excluded from the study. The study's primary outcome was the complete resolution of reflux in the renal unit upon radiological evaluation (specifically a voiding cystourethrogram (VCUG)) performed after STING. In investigating the effect of parameters age, gender, side (right or left), reflux level (low, moderate, high), LUTD history, UDR, DRF and presence of reflux in the other unit in predicting resolution after the first dextranomer/hyaluronic acid copolymer acid injection, we carried out a renal unit-based evaluation. Therefore, we had the chance to examine the effects of different features between units on resolution in bilateral cases where resolution was observed in one unit but not in the other.

In the preoperative evaluation, all patients underwent laboratory analysis, urine ultrasonography, VCUG and dimercaptosuccinic acid (DMSA) screening. VCUG grading was done according to the system defined by the International Reflux Study Committee^17^. In determining the reflux level according to the VCUG results of the patients, grade 1–2 reflux was defined as low, grade 3 reflux was defined as moderate, and grade 4–5 reflux was defined as high^18^. The receiver operating characteristic (ROC) analysis results to determine the DRF parameter cut-off value were insignificant. For this reason, the DRF cut-off value was chosen to be 45, the smallest value of the normal range. In the literature, the normal value of DRF is accepted to be between 45–55%^19–21^. The VCUG image was used to calculate the UDR. The largest ureter diameter in the false pelvis, defined as the area below the highest point of the iliac crest, was calculated in millimetres (mm). This calculated number was then divided by the distance from the bottom of the L1 vertebral body to the top of the L3 vertebral body to control patient size and radiographic magnification^22^. LUTD was accepted as the presence of lower urinary tract symptoms such as urgency, wetting (> 5 years), constipation, poor voiding flow rate, and pollakiuria, which can be determined by symptom scores or urodynamic studies in toilet-trained children. Patients with LUTD were primarily treated for LUTD. Behavioural adjustments and/or medical therapy were used in the treatment. Adhering to the European Association of Urology (EAU) and European Society of Pediatric Urology (ESPU) guidelines, all patients underwent complete re-evaluation after LUTD treatment.

The diagnosis of VUR and defining the risk group of the patients were performed according to the EAU/ESPU guidelines. VUR was diagnosed after various conditions, such as prenatal hydronephrosis, a history of febrile urinary tract infection, sibling reflux, or renal scarring shown on a DMSA scan. EAU/ESPU guidelines were followed when deciding on follow-up and treatment protocols. Preferably, all patients were started on antibiotic prophylaxis. Prophylaxis was given as a single oral dose at night (a third or a fourth of the usual dose). Trimethoprim–sulfamethoxazole and amoxicillin were the antibiotics used for prophylaxis. In the first two years of life, regardless of the grade of reflux and the presence of renal scarring, surgical treatment was not performed unless there was a breakthrough febrile urinary tract infection. Indications for STING were recurrent febrile urinary tract infection under antibiotic prophylaxis, renal scarring, persistent high-grade reflux, and sometimes parental requests. Breakthrough febrile urinary tract infection was defined as fever above 38 °C, the presence of symptoms (urinary urgency, dysuria, back pain, mostly in older children), and at least growth of 100,000 colony-forming units/ml in urine culture (middle-stream urine or obtained from the bag).

The STING procedure was performed on all the patients under general anaesthesia by/under the supervision of a senior pediatric urologist. Antibiotic prophylaxis was administered to all the patients. Dextranomer/hyaluronic acid copolymer was used in all the patients in varying volumes, depending on the orifice structure of the patient. Diagnostic cystoscopy was performed with a pediatric cystoscope. Orifices were evaluated. Next, the needle was inserted submucosally in the orifice at the six o’clock position, and the material was injected until a slit-like orifice was seen with mounding of the ureteral orifice. We performed control ultrasonography one month and control VCUG three months after the procedure. We continued antibiotic prophylaxis until a control VCUG was performed. We included the patients who did not have reflux based on the control VCUG performed three months later in the resolution ( +) group and the patients with any degree of reflux in the resolution (−) group. We defined the resolution (−) group as the “persistence of reflux after STING” group. The Strobe Statement was adhered to while writing the manuscript. Data were analyzed with IBM SPSS V23. ROC analysis was used to determine the cut-off values of quantitative parameters to predict reflux resolution after STING. A Pearson chi-square test and Yates correction were used to compare the categorical data according to the groups, and multiple comparisons of the results were analyzed using a Bonferroni corrected Z test. Factors affecting reflux resolution were analyzed using binary logistic regression analysis. Analysis results were presented as frequency (percentage) for categorical data. The significance level was determined to be p < 0.050.

## Results

Once patients who met at least one of the exclusion criteria were excluded, 127 of 199 patients were eligible for the study. Of the 199 patients, 22 patients had a history of vesicoureteral reflux surgery (2 patients had a history of ureteroneocystostomy, 20 patients had a history of STING), 30 patients had an accompanying pathology (neurogenic bladder, ureteropelvic junction obstruction, double collecting system, PUV, or spinal deformity), and 20 patients were excluded due to missing data. The STING procedure was performed on 180 renal units of 127 patients. Of the patients, 34 (26.7%) were male and 93 (73.3%) were female; their mean age was 74.62 ± 46.18 (3–193) months; and 53 were affected bilaterally and 74 were affected unilaterally. Eighty-seven renal units were right-sided, and 93 renal units were left-sided. Postoperative fever developed in two patients. After parenteral antibiotic therapy, the patients recovered and were discharged. Upper urinary tract and ureteral dilatation due to ureteral stenosis after injection was not observed in any patient. Since the study was designed based on control VCUG findings approximately three months after the operation, it includes early postoperative results. As a result of the unit-based evaluation, reflux resolution was observed in 106 of 180 renal units in the control VCUG performed 3 months later.

**Figure 1:**
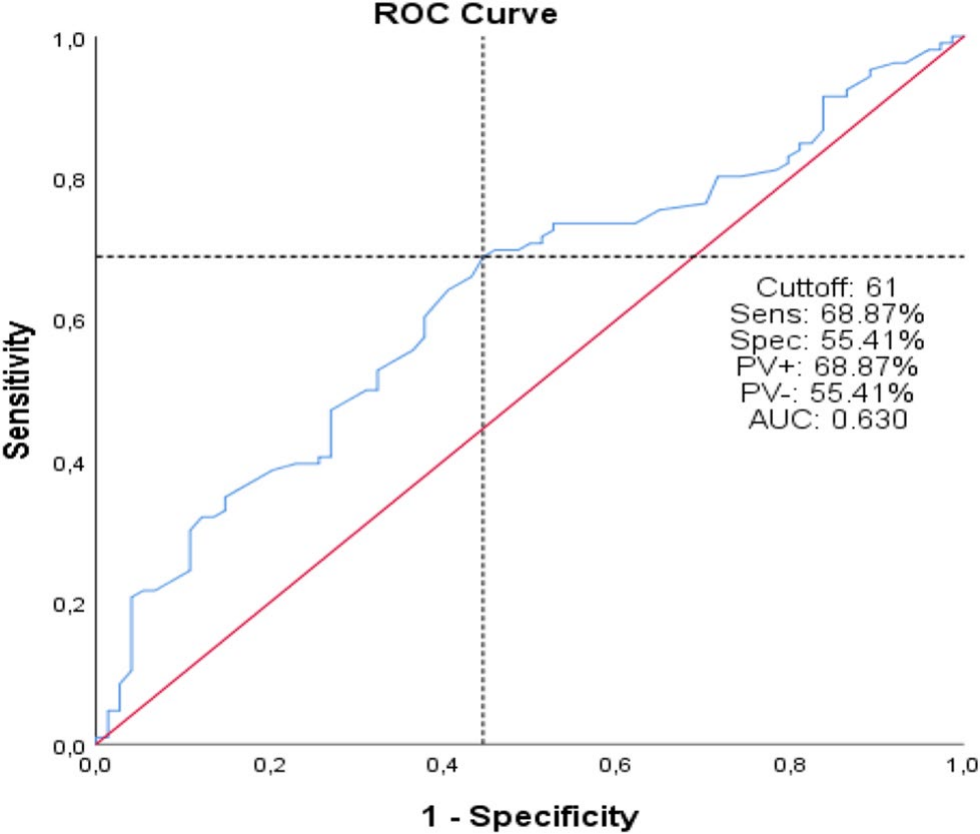
ROC curve of Age parameter in predicting reflux resolution after STING.

**Figure 2:**
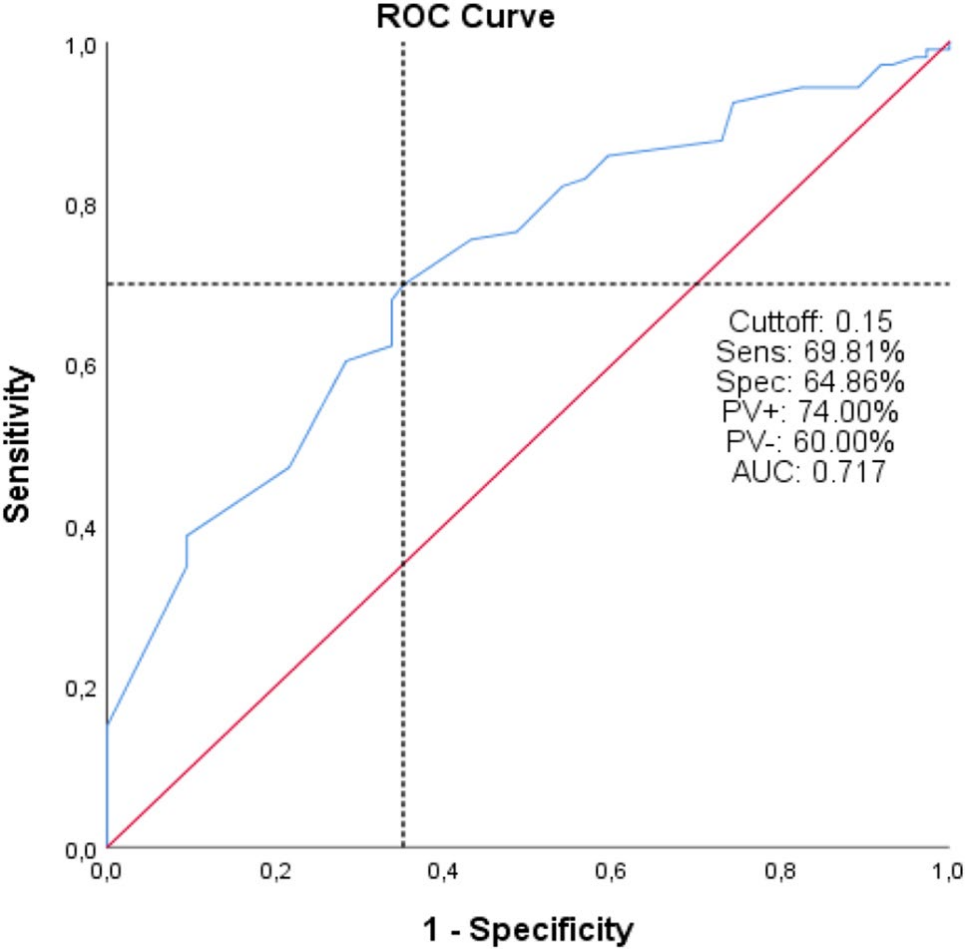
ROC curve of UDR parameter in predicting reflux resolution after STING.

As a result of the ROC analysis performed to determine the cut-off values of age and UDR parameters, the cut-off value for the age parameter was 61 months (AUC: 0.630 (0.548–0.711), 95% CI), and the cut-off value for the UDR parameter was determined to be 0.15 (AUC: 0.717 (0.643–0.791), 95% CI) (Fig. 1 and Fig. 2). Age < 61 months (univariate: p = 0.001, multivariate: p = 0.015, HR: 2.352 (1.181–4.686), OR (95% CI)), moderate reflux level (grade 3) (univariate: p < 0.001, multivariate: p = 0.019, HR: 2.703 (1.177–6.209), OR (95% CI)), DRF (differential renal function) < 45 (univariate: p = 0.020, multivariate: p = 0.047, HR: 1.992 (1.009–3.935), OR (95% CI)), and UDR (ureteral diameter ratio) > 0.15 (univariate: p < 0.001, multivariate: p = 0.005, HR: 2.786 (1.368–5.672), OR (95% CI)) were found predictors of reflux persistence after STING surgery both univariate and multivariate analysis (Table 1, Table 2). High reflux level (grade 4–5) was statistically significant in univariate analysis (p < 0.001) but not statistically significant in multivariate analysis (p = 0.215) (Table 1, Table 2).

**Table 1:** Parameter comparisons by reflux resolution rates after STING.

	Resolution (−) n (%)	Resolution ( +) n (%)	Test statistics	p
Age (Month)				
< 61	41 (55.4)	33 (31.1)	10.606	**0.001**^**c**^
≥ 61	33 (44.6)	73 (68.9)		
Gender				
Male	17 (23)	26 (24.5)	0.004	0.950^d^
Female	57 (77)	80 (75.5)		
Side of reflux				
Right	36 (48.6)	51 (48.1)	0.005	0.944^c^
Left	38 (51.4)	55 (51.9)		
Reflux level				
Low (Grade 1–2)	22 (29.7)a	64 (60.4)b	16.459	** < 0.001**^**c**^
Moderate (Grade 3)	26 (35.1)a	20 (18.9)b		
High (Grade 4–5)	26 (35.1)a	22 (20.8)b		
DRF				
< 45	38 (51,4)	36 (34)	5.443	**0.020**^**c**^
≥ 45	36 (48,6)	70 (66)		
UDR				
≤ 0,15	26 (35,1)	74 (69,8)	21.222	** < 0.001**^**c**^
> 0,15	48 (64,9)	32 (30,2)		
Low urinary tract dysfunction				
Yes	23 (31,1)	29 (27.4)	0.141	0.708^d^
No	51 (68,9)	77 (72.6)		
Presence of reflux in the other unit				
Yes	45 (25,0)	61 (33.9)	0.032	0.859^d^
No	29 (27,4)	45 (25.0)		

a,b: No difference between groups with same character. Frequency (percent).

^c^Pearson chi-square test, ^d^Yates correction.

Significant values are in bold.

**Table 2:** Examining the predictors of reflux persistence after STING with Binary Logistic Regression Analysis.

	Univariate		Multivariate	
	OR (%95 CI)	p	OR (%95 CI)	p
Age (Month)				
< 61	2.748 (1.485—5.087)	**0.001**	2.352 (1.181—4.686)	**0.015**
> = 61	Reference			
Reflux level				
Low (Grade 1–2)	Reference			
Moderate (Grade 3)	3.782 (1.772—8.07)	**0.001**	2.703 (1.177—6.209)	**0.019**
High (Grade 4–5)	3.438 (1.63—7.252)	**0.001**	1.71 (0.733—3.989)	0.215
DRF				
< 45	2.052 (1.118—3.769)	**0.020**	1.992 (1.009—3.935)	**0.047**
> = 45	Reference			
UDR				
< = 0.15	Reference			
> 0.15	4.269 (2.269—8.034)	** < 0.001**	2.786 (1.368—5.672)	**0.005**

Significant values are in bold.

## Discussion

Endoscopic injection is frequently preferred for patients with vesicoureteral reflux because it is minimally invasive, reproducible, associated with a short hospital stay, and has a faster learning curve compared to open surgery^8^. However, the gold standard method is still reconstructive surgery (open and minimally invasive) because of its reliability, low complication rate, and high reflux resolution rates (92%–98%) after surgery^23^. Among patients receiving endoscopic injections, those with persistent reflux will either need repeated injections or will have persistent reflux despite repeated injections and will need reconstructive surgery. Many studies in the literature investigated the effects of various factors on reflux resolution after endoscopic injection. However, there is still a need for studies in which many different factors are examined together.

In this study, we aimed to examine the effect of various parameters on reflux resolution after STING. As we performed a unit-based evaluation in this study, we did not use the term “surgical success” when evaluating our results. Instead, we preferred to use the terms “reflux resolution after STING” or “persistence of reflux”. There is no study in the literature examining the effects of DRF and UDR parameters together on reflux resolution after STING. In multivariate logistic regression analysis, both parameters were found to have an effect on reflux resolution after STING. Therefore, due to their combined evaluation, we discovered that both parameters are independent predictive factors.

In a 2006 meta-analysis of renal unit-based evaluation, the reflux resolution rate after STING was 75.7%. In our renal unit-based evaluation, the reflux resolution rate after STING was 58.8%. In a subgroup analysis of studies in which it is appropriate to evaluate resolution after STING according to VUR grade, the grade 1–2 resolution rate was 78.5%, the grade 3 resolution rate was 72%, the grade 4 resolution rate was 63%, and the grade 5 resolution rate was 51%. In the subgroup analysis, those with high-grade reflux (grade 4–5) comprised 15.9% of all patients. In our study, renal units with high-level reflux (grade 4–5) comprised 26.6% of all renal units. One reason our resolution rates after STING are lower than in the meta-analysis may be that the percentage of renal units with high-level reflux was higher in our study than in the studies included in the meta-analysis (26.6% & 15.9%). In a group of studies included in the meta-analysis, monitoring of grade 1 reflux in the postoperative VCUG was also considered as success. In our study, we included renal units with grade 1 VUR in the postoperative VCUG in the “reflux persistence after STING” group. Perhaps our resolution rates after STING would have been higher if we had not included renal units with grade 1 VUR detected in the postoperative VCUG in the reflux persistence group^8^.

Dogan et al. (2015) found that being older than 54 months had a positive effect on resolution rates after endoscopic injection^18^, Şahan et al. (2015) found that being older than 60 months had a positive effect on resolution rates after endoscopic injection^24^, and Ceylan et al. (2021) found that being older than 84 months had a positive impact on resolution rates after endoscopic injection^25^. Our study found that being < 61 months had a negative impact on reflux resolution after STING. Although the age limit in our study was different from that in the articles in the literature, similar to the articles in the literature, being higher than the cut-off value determined for age was found to be a predictor for the persistence of reflux.

In a study conducted in 2020, the effects of different VCUG parameters, such as the UDR, reflux timing (early filling, filling, voiding), and delay in the removal of upper urinary tract contrast, on the reflux resolution rates after STING were examined. Two hundred patients and 248 renal units were evaluated. If the UDR was > 0.24, the probability of reflux persistence was 3.076 times higher^12^ In a study conducted in 2014, the value of UDR in predicting the reflux persistence after STING was investigated in children with grade 3–4 reflux. High UDR was found to be a factor that affected reflux persistence after endoscopic surgery^13^ In a study conducted in 2019, the effect of UDR on spontaneous resolution and post-endoscopic surgery resolution rates was examined. It was observed that the UDR averages increased as the reflux degree increased and that the UDR was lower in patients whose reflux spontaneously resolved. The results of endoscopic injections in 451 renal units of 248 patients were analyzed. The UDR was found to be higher in those who had reflux persistence after surgery^14^ Similar to these studies, in our study the fact that the UDR was greater than the cut-off value of 0.15 was found to be a factor that increased the chance of failure.

In a study conducted in 2007, a hypoplastic nephroureteral system was found to be a factor that reduced reflux resolution rates after endoscopic injection in patients with grades 3, 4, and 5 reflux^15^ In a long-term study conducted in 2011, the data of patients with recurrent reflux after successful STING were examined. Renal function < 40 on a DMSA scan was not found to be effective in either STING success or recurrence after successful STING^16^ In our study, DRF < 45 was found to be a factor that increased reflux persistence after STING. There is insufficient literature on this subject, and studies with more extensive patient data are needed.

According to a bibliometric review conducted in 2021, between 2005 and 2019 14 studies that included patients who had endoscopic injections due to VUR and that examined the factors affecting reflux resolution rates after endoscopic injection were evaluated. Although bilaterality was found to be an influential factor in endoscopic failure in univariate analysis in two studies^26,27^ included in this bibliometric review, it was not seen to be an influential factor in any study in multivariate analysis. Similar to this meta-analysis, in our study, the presence of reflux in the other unit was found not to affect the persistence of reflux. There was heterogeneity in the studies included in this bibliometric review regarding, for example, the evaluation, diagnosis, and treatment of LUTD. Some studies found that LUTD affects reflux resolution after surgery, while others found the opposite^11^ In our study, we found that having a history of LUTD did not affect reflux resolution after STING. All patients with preoperative LUTD were treated for LUTD before surgery. However, data on what percentage of patients’ LUTD could be completely cured before the procedure are unavailable, as in many studies included in the meta-analysis. This is a limiting factor in our study.

Other techniques, such as the hydrodistention implantation technique (HIT) and double HIT, began to be implemented in our clinic in the following years. However, this study only included patients who underwent STING. Patients who underwent HIT and double HIT could not be included in this study due to confusion in the operation notes and data about which technique was applied. If these patients had been included in the study, perhaps our reflux resolution rate after STING would have been different. We used hyaluronic acid/dextranomer copolymer material for all patients. There are times when this material cannot be obtained, so we have to perform ureteroneocystostomy on patients for whom STING is suitable. If the material had been available and STING had been used for these patients, perhaps our reflux resolution rate after STING would have been different. In addition, we do not have data on injected material volumes. These are other limiting factors in our study. We tend to follow patients who choose to continue their follow-up in our clinic, until puberty. This study's follow-up period is limited to the control VCUG taken at the postoperative third month. We could not examine reflux recurrence in the long-term follow-up of patients with reflux resolution in the postoperative VCUG. Our clinic is a reference clinic where patients come from the surrounding provinces. Patients referred from different provinces may prefer to continue their follow-up in their own city after reflux resolution. Therefore, our data is not suitable for examining long-term results. We could not provide the results of the second or third STING procedure for some patients, both because of insufficient data and because we designed our study according to early postoperative results. The lack of long-term follow-up results or lack of data for patients who underwent a second or third STING procedure and the retrospective nature of our study are also limiting factors in our study.

In the present study, the effects of UDR and DRF parameters on reflux persistence after STING were evaluated together. To our knowledge, there is no study in the literature where UDR and DRF parameters are evaluated together. UDR > 0.15 and DRF < 45 were found to be independent risk factors of reflux persistence after STING. Other independent risk factors were moderate reflux level (grade 3) and age < 61 months. Although not an independent risk factor, the presence of high-level reflux (grade 4–5) was also found to be a risk factor for the persistence of reflux after STING. Parents of patients with this profile should be told that although this method is much less invasive than ureteroneocystostomy, there may be a need for a second or third injection. For the patients themselves, factors such as the families’ wishes, the families’ compliance with the treatment process, and the families’ access to health services should be taken into consideration, as ureteroneocystostomy may be a better option than repetitive endoscopic interventions. Therefore, to fully understand which patient group will not benefit from STING, there is a need for a randomized prospective multicenter series with a larger patient group that examines many different parameters, including UDR and DRF, followed by a nomogram study.

## Data Availability

The datasets used and/or analysed during the current study available from the corresponding author on reasonable request.
